# MNP-Enhanced Microwave Medical Imaging by Means of Pseudo-Noise Sensing

**DOI:** 10.3390/s21196613

**Published:** 2021-10-04

**Authors:** Sebastian Ley, Jürgen Sachs, Bernd Faenger, Ingrid Hilger, Marko Helbig

**Affiliations:** 1 Biosignal Processing Group, Technische Universität Ilmenau, 98693 Ilmenau, Germany; marko.helbig@tu-ilmenau.de; 2 Electronic Measurements and Signal Processing Group, Technische Universität Ilmenau, 98693 Ilmenau, Germany; juergen.sachs@tu-ilmenau.de; 3 ILMSENS GmbH, 98693 Ilmenau, Germany; 4 Institute of Diagnostic and Interventional Radiology, Jena University Hospital, Friedrich Schiller University Jena, 07747 Jena, Germany; bernd.faenger@uni-jena.de (B.F.); ingrid.hilger@med.uni-jena.de (I.H.)

**Keywords:** ultra-wideband, magnetic nanoparticles, microwave imaging, medical imaging, magnetic modulation, M-sequence radar technology

## Abstract

Magnetic nanoparticles have been investigated for microwave imaging over the last decade. The use of functionalized magnetic nanoparticles, which are able to accumulate selectively within tumorous tissue, can increase the diagnostic reliability. This paper deals with the detecting and imaging of magnetic nanoparticles by means of ultra-wideband microwave sensing via pseudo-noise technology. The investigations were based on phantom measurements. In the first experiment, we analyzed the detectability of magnetic nanoparticles depending on the magnetic field intensity of the polarizing magnetic field, as well as the viscosity of the target and the surrounding medium in which the particles were embedded, respectively. The results show a nonlinear behavior of the magnetic nanoparticle response depending on the magnetic field intensity for magnetic nanoparticles diluted in distilled water and for magnetic nanoparticles embedded in a solid medium. Furthermore, the maximum amplitude of the magnetic nanoparticles responses varies for the different surrounding materials of the magnetic nanoparticles. In the second experiment, we investigated the influence of the target position on the three-dimensional imaging of the magnetic nanoparticles in a realistic measurement setup for breast cancer imaging. The results show that the magnetic nanoparticles can be detected successfully. However, the intensity of the particles in the image depends on its position due to the path-dependent attenuation, the inhomogeneous microwave illumination of the breast, and the inhomogeneity of the magnetic field. Regarding the last point, we present an approach to compensate for the inhomogeneity of the magnetic field by computing a position-dependent correction factor based on the measured magnetic field intensity and the magnetic susceptibility of the magnetic particles. Moreover, the results indicate an influence of the polarizing magnetic field on the measured ultra-wideband signals even without magnetic nanoparticles. Such a disturbing influence of the polarizing magnetic field on the measurements should be reduced for a robust magnetic nanoparticles detection. Therefore, we analyzed the two-state (ON/OFF) and the sinusoidal modulation of the external magnetic field concerning the detectability of the magnetic nanoparticles with respect to these spurious effects, as well as their practical application.

## 1. Introduction

Magnetic nanoparticles (MNPs) are of great importance for a variety of biomedical applications. They can be used to target delivery of multiple immunotherapeutics [1], as well as for accurate targeting of therapeutic-drug-delivery microrobots [2]. Magnetic field hyperthermia offers a promising approach to support cancer therapies in which the MNPs are locally heated by a time-varying magnetic field [3]. Furthermore, magnetoelastic biosensors in combination with magnetic nanoparticles offer an approach for the wireless detection of pathogens in liquids and for real-life diagnostic purposes [4].

The approach of modulating magnetic nanoparticles by a magnetic field to improve sensitivity has been used for different biomedical applications such as a computational cytometer based on magnetically modulated lensless speckle imaging [5] and magnetomotive photoacoustic (mmPA) imaging [6]. Magnetically modulated optical nanoprobes that emit fluorescent signals in response to rotating magnetic fields promise to improve immunoassays and intracellular chemical recognition [7].

Furthermore, the possibility of selective MNP targeting to a region of interest (e.g., tumor) makes them attractive as a contrast agent for diagnostics and imaging. Therefore, the need for MNP imaging methods has motivated research in several technologies such as magnetic resonance imaging (MRI) [8], magnetic particle imaging (MPI) [9], magnetorelaxometry (MRX) [10], and microwave imaging (MWI) [11,12]. The usage of MNPs (iron oxide nanoparticles) in MRI influences the longitudinal relaxation time T1 or the transverse relaxation time T2. If a sufficient amount of MNPs accumulates in the tissue, the spin–spin relaxation time T2 will decrease, as well as the signal intensity, which results in a negative contrast enhancement [8,13]. Further investigations demonstrated that ultrasmall spherical iron oxide nanoparticles (<5 nm) reduce the longitudinal relaxation time T1 and act as a positive contrast agent for MRI [14]. Magnetic particle imaging is a noninvasive and quantitative three-dimensional (3D) imaging modality of ferromagnetic nanoparticles that offers high spatial and temporal resolution [9]. The approach relies on the nonlinear magnetization curve of the ferromagnetic nanoparticles. Magnetorelaxometry is a noninvasive method, which offers qualitative and quantitative information about the spatial distribution of MNPs [15,16]. The approach is based on the detection of the time-varying magnetic flux density of the MNPs.

**Figure 1:**
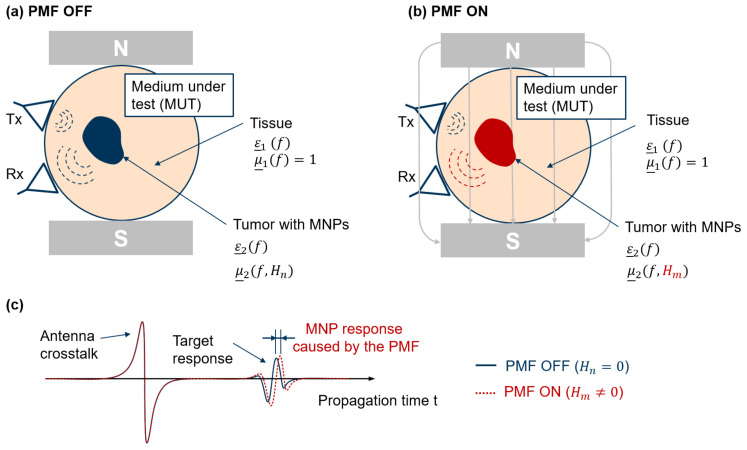
Illustration of the contrast-enhanced MWI scenario for an ON/OFF modulation of the external PMF (**a**) without the presence of a PMF and (**b**) with the presence of a PMF; (**c**) resulting IRFs for both PMF states.

Our method is based on ultra-wideband (UWB) differential microwave imaging (MWI). The approach is that functionalized MNPs are injected intravenously and that these particles are able to bind specifically to the tumorous tissue. Figure 1 shows the measurement principle for the case that a sufficient amount of MNPs has accumulated within the cancerous tissue. It is illustrated exemplarily for one channel, which is the combination of a transmitting and a receiving antenna. The transmitting antenna (Tx) emits low-power electromagnetic waves in the microwave frequency range into the medium under test (MUT). The electromagnetic waves propagate according to the constitutive parameters ($\underline{\varepsilon },\underline{\mu }$) of the MUT and are partially reflected at each dielectric and magnetic boundary. Figure 1c illustrates the impulse response function (IRF) measured by the receiving antenna (Rx) corresponding to the scenario without and with the presence of an external polarizing magnetic field (PMF). For the sake of clarity, the antenna crosstalk and the target response are separated in the diagram (see Figure 1c), which is not the case in a real measurement. Furthermore, the direct antenna crosstalk is much higher than the expected tumor response. In order to eliminate the undesired signal components and to separate the MNP response, a differential measurement between the OFF and ON state of the PMF is performed. If a sufficient amount of MNPs binds to the tumor, these MNPs can be modulated by the PMF, which leads to a changing scattering behavior due to the change in relative permeability $\underline{\mu }$. Due to the nonmagnetic properties of the tissue, the surrounding medium is not affected by the PMF, and therefore, the changes in the measured signal result from the presence of MNPs, as illustrated in Figure 1c.

In this paper, we investigate different parameters related to the detectability of MNPs. In the first experimental setup, we analyze the MNP response depending on the magnetic field intensity of the PMF, as well as the influence of the viscosity of the MNPs and the surrounding medium in which the particles are embedded, respectively.

In the second experiment, we investigate the influence of the target position (depth of the target) on the detectability and the 3D imaging results in a real measurement setup. In this context, we also consider the influence of the PMF on our measurement results even without the presence of MNPs. Such a disruptive influence can be caused by interactions between the PMF and the MWI system or be provoked by hidden magnetic materials in the measurement setup, as investigated by Bucci et al. [17]. Moreover, we present a compensation approach of the inhomogeneity of the magnetic field. In addition, we enhanced our MWI system in order to perform imaging with an ON/OFF and a sinusoidal modulation of the PMF, as described in our previous work [12,18]. The detection and imaging results of both modulation types are discussed concerning a practical application.

## 2. Materials and Methods

### 2.1. Magnetic Susceptibility of MNPs in the Microwave Frequency Range

The frequency-dependent complex magnetic susceptibility $\underline{\chi }\left(f\right)=\chi^{\prime }\left(f\right)-i\chi^{\prime \prime }\left(f\right)$ of single-domain MNPs dispersed in a fluid can be described by [19]:

(1) $$\underline{\chi }\left(f\right)=\underline{\mu }\left(f\right)-1=\frac{1}{3}\left({\underline{\chi }}_{\|}\left(f\right)+2{\underline{\chi }}_{⊥}\left(f\right)\right)$$

with the parallel susceptibility ${\underline{\chi }}_{\|}$ and the perpendicular susceptibility ${\underline{\chi }}_{⊥}$ depending on the frequency *f*. The parallel susceptibility is mainly related to the Néel and Brownian relaxations. If we assume monodispersed MNPs, it can be expressed by the Debye equation:

(2) $${\underline{\chi }}_{\|}\left(f\right)=\frac{\chi_{\|0}}{1+i\;2\pi f\tau_{\|}}$$

with the static parallel susceptibility $\chi_{\|0}$ and the effective relaxation time $\tau_{\|}$, a time constant that describes the reorientation of the MNP due to the change of an external magnetic field. If both relaxation mechanisms act simultaneously, the effective relaxation time is given by:

(3) $$\tau_{\|}=\frac{\tau_{n}\cdot \tau_{b}}{\tau_{n}+\tau_{b}}$$

with the Néel relaxation time:

(4) $$\tau_{n}=\tau_{0}\exp\left(\frac{K_{a}V_{p}}{k_{b}\vartheta }\right)$$

and the Brownian relaxation time:

(5) $$\tau_{b}=\frac{3V_{h}\eta }{k_{b}\vartheta }$$

where $\tau_{0}$ is a characteristic time, $K_{a}$ the anisotropy factor, $V_{p}$ the core volume of the particle, $k_{b}$ the Boltzmann constant, ϑ the absolute temperature, $V_{h}$ the hydrodynamic volume of each MNP, and η the effective viscosity of the carrier liquid [20,21].

The magnetic susceptibility in the gigahertz range is mainly determined by the transverse or perpendicular susceptibility, which is related to the ferromagnetic resonance and can be described by:

(6) $${\underline{\chi }}_{⊥}\left(f\right)=\chi_{⊥0}\frac{1+i\;2\pi f\tau_{2}+\Delta }{\left(1+i\;2\pi f\tau_{2}\right)\left(1+i\;2\pi f\tau_{⊥}\right)+\Delta }$$

where $\chi_{⊥0}$ is the static transverse susceptibility, $\tau_{⊥}$ is the transverse magnetic relaxation time, and $\tau_{2}$ a second effective relaxation time, and:

(7) $$\Delta =\frac{\sigma \tau_{2}\left(\tau_{n}-\tau_{⊥}\right)}{\alpha^{2}\tau_{n}^{2}}$$

where σ is the ratio of anisotropy energy to thermal energy, $\tau_{n}$ the Néel relaxation time, and α a dimensionless damping factor [22,23,24].

If we assume equilibrium conditions, the magnetic moment and the anisotropy axis of a particle will be parallel. The application of an electromagnetic microwave field leads to a precession of the magnetic moment about the easy axis. In case the angle between the easy axis and the magnetic moment is small, the resonance frequency is given by:

(8) $$f_{res}=\frac{\gamma H_{A}}{2\pi }$$

where γ is the gyromagnetic ratio and $H_{A}$ the internal field. Considering a single-domain particle with an uniaxial anisotropy, the internal anisotropy field is defined by:

(9) $$H_{A}=\frac{2K_{a}}{M_{s}}$$

with the saturation magnetization $M_{s}$ of the MNP core [19]. Finally, the overall frequency-dependent susceptibility can be described by combining Equations (1), (2) and (6) corresponding to [25]:

(10) $$\underline{\chi }\left(f\right)=\frac{1}{3}\left[\frac{\chi_{\|0}}{1+i\;2\pi f\tau_{\|}}+2\chi_{⊥0}\frac{1+i\;2\pi f\tau_{2}+\Delta }{\left(1+i\;2\pi f\tau_{2}\right)\left(1+i\;2\pi f\tau_{⊥\;eff}\right)+\Delta }\right]$$

where $\tau_{⊥}$ is replaced by:

(11) $$\tau_{⊥\;eff}=\frac{\tau_{⊥}\cdot \tau_{b}}{\tau_{⊥}+\tau_{b}}$$

in order to include the effects of the Brownian relaxation. If an additional external PMF with an amplitude *H* is applied, the resonance frequency will increase corresponding to:

(12) $$f_{res}\left(H\right)=\frac{\gamma (H_{A}+H)}{2\pi }$$

as described by Fannin et al. [26]. Furthermore, the dependence of the static susceptibilities and the relaxation times on the magnetic field intensity have to be taken into account [25,27].

The magnetic susceptibility of MNPs depending on the magnetic field intensity of an external PMF was measured by Fannin et al. [19] in the frequency range between 30 MHz and 6 GHz for magnetic field intensities between 0 kA/m and 116 kA/m and by Bucci et al. [28] in the frequency range between 100 MHz and 8 GHz for magnetic field intensities between 0 kA/m and 160 kA/m. The results of these investigations are fundamental for MNP detection and imaging by means of UWB technology, as this change of the magnetic susceptibility $\underline{\chi }\left(f,H\right)$ or relative permeability $\underline{\mu }\left(f,H\right)$ of the MNPs, caused by the changing external magnetic field intensity, results in a change of the measured UWB differential signal.

The principle of the scenario is shown in Figure 1, whereby the resulting IRFs (Figure 1c) can be described by two components corresponding to:

(13) $$y\left(t,H\right)=y_{cl}\left(t\right)+y_{tar}\left(t,H\right)$$

with the propagation time *t*, the magnetic field intensity *H*, the static clutter components $y_{cl}\left(t\right)$ (e.g., antenna crosstalk), and $y_{tar}\left(t,H\right)$ representing the target response. Under some idealized conditions (no drift, noise, and spurious effects caused by the PMF), the received target response can be modeled as suggested by Sachs et al. [29]:

(14) $$y_{tar}\left(t,H\right)=y_{0}\left(t\right)*\Gamma \left(t,H\right)$$

where the symbol ∗ represents the convolution and $y_{0}\left(t\right)$ summarizes all magnetic-field-independent components of the target reflection (including, e.g., path-dependent attenuation and propagation time delay). The magnetic-field-dependent reflection coefficient Γ(t,H) describes the signal reflection at the boundary between the surrounding tissue and the MNP-loaded tumor. The static clutter $y_{cl}\left(t\right)$ can be eliminated by a differential measurement:

(15) $$\begin{array}{cc} \Delta y(t,\Delta H_{m,n}) & =y\left(t,H_{m}\right)-y\left(t,H_{n}\right)\\ \; & =y_{0}\left(t\right)*\left[\Gamma \left(t,H_{m}\right)-\Gamma \left(t,H_{n}\right)\right]. \end{array}$$

In order to demonstrate the dependency of the measured UWB signals on the magnetic susceptibility of the MNPs, Equation (15) is converted to the frequency domain:

(16) $$\Delta \underline{Y}\left(f,\Delta H_{m,n}\right)={\underline{Y}}_{0}\left(f\right)\cdot \left[\underline{\Gamma }\left(f,H_{m}\right)-\underline{\Gamma }\left(f,H_{n}\right)\right].$$

For the sake of simplicity, we assume a plane wave propagation, as well as a specular reflection at the boundary between the host medium and the target. Thus, the reflection coefficient can be formulated by:

(17) $$\underline{\Gamma }\left(f,H\right)=\frac{{\underline{Z}}_{2}\left(f,H\right)-{\underline{Z}}_{1}\left(f\right)}{{\underline{Z}}_{2}\left(f,H\right)+{\underline{Z}}_{1}\left(f\right)}.$$

Furthermore, the impedance of the healthy tissue can be described by:

(18) $${\underline{Z}}_{1}\left(f\right)=Z_{0}\cdot \sqrt{\frac{{\underline{\mu }}_{1}\left(f\right)}{{\underline{\varepsilon }}_{1}\left(f\right)}}$$

with ${\underline{\mu }}_{1}\left(f\right)=1$ due to the nonmagnetic properties of tissue and $Z_{0}=\sqrt{\mu_{0}/\varepsilon_{0}}$, where $\mu_{0}$ is the permeability constant in free space and $\varepsilon_{0}$ is the permittivity constant in free space. The impedance of the tumor tissue with MNPs can formulated by:

(19) $${\underline{Z}}_{2}\left(f\right)=Z_{0}\cdot \sqrt{\frac{{\underline{\mu }}_{2}\left(f,H\right)}{{\underline{\varepsilon }}_{2}\left(f\right)}}.$$

Finally, the reflection coefficient is given by the combination of Equations (17)–(19), resulting in:

(20) $$\underline{\Gamma }\left(f,H\right)=\frac{\sqrt{{\underline{\mu }}_{2}\left(f,H\right)\cdot \frac{{\underline{\varepsilon }}_{1}\left(f\right)}{{\underline{\varepsilon }}_{2}\left(f\right)}}-1}{\sqrt{{\underline{\mu }}_{2}\left(f,H\right)\cdot \frac{{\underline{\varepsilon }}_{1}\left(f\right)}{{\underline{\varepsilon }}_{2}\left(f\right)}}+1}.$$

Considering this equation, it is obvious that the reflection coefficient $\underline{\Gamma }\left(f,H\right)$ depends on the dielectric properties ${\underline{\varepsilon }}_{1}$ and ${\underline{\varepsilon }}_{2}$, as well as on the magnetic susceptibility $\underline{\chi }={\underline{\mu }}_{2}-1$ of the investigated tissues.

### 2.2. UWB M-Sequence Radar

The UWB radar systems used in this work were based on a correlation measurement method (M-sequence) developed at Technische Universität Ilmenau. The technique determines the IRF of the MUT by computing the cross-correlation between the stimulus signal (M-sequence) and the recorded signal. The stimulus is generated by a binary high-speed shift register and a single-tone RF clock, whereby the clock rate defines the bandwidth of the radar system. The signal energy of the sounding wave is distributed over the measurement time. This ensures a low-voltage exposure of the MUT, which enables the technology for medical applications. In addition, the M-sequence approach can be applied to realize flexible and robust (low jitter and drift) UWB radar systems, as presented by Sachs [30].

### 2.3. Investigation of the MNP Response Depending on the Viscosity and the Magnetic Field Intensity

**Figure 2:**
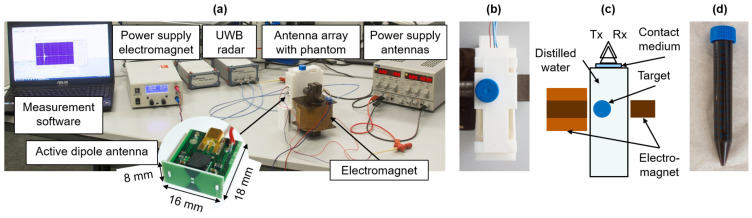
Experimental measurement setup: (**a**) A 3D-printed tank filled with distilled water is placed inside the air gap of the electromagnet. This tank acts as an antenna array. The active dipole antennas are connected to an UWB M-sequence radar. (**b**) Top view of the phantom inside the air gap of the electromagnet with a mount holding the test glass in the right position. (**c**) Corresponding schematic of the phantom box inside the air gap. (**d**) One of the test tubes filled with MNPs embedded in gelatin.

Figure 2 shows the measurement setup for examining the influence of both the magnetic field strength and the medium in which the MNPs were embedded on the MNP response. The experimental setup was based on our previous work [31]. In particular, it consisted of a 3D-printed box (60×38×100 mm${}^{3}$) filled with distilled water. The box was placed in the air gap of the electromagnet and acted as holder for the targets, as well as for the antennas. In order to improve the MNP response, the 3D-printed box was modified compared to our previous work. The wall thickness of the box in front of the antennas was reduced to ensure a better penetration of the electromagnetic waves into the MUT. The active dipole antennas were connected to an UWB radar system via nonmagnetic high-frequency cables.

The targets consisted of a test glass filled with MNPs (WHKS 1S12, Liquids Research Limited, Bangor, UK) diluted in distilled water or embedded in agar, gelatin, or two types of oil–gelatin. The noncoated MNPs had a saturation magnetization of $M_{s}=400$ G and a particle diameter of 10 nm. The viscosity of the raw ferrofluid was η<50 cP. We prepared fifteen targets (three of each medium) with a volume of 15 mL including 100 mg of MNPs. The agar targets consisted of 97 wt./wt.% distilled water mixed with the MNPs and 3 wt./wt.% agar. The gelatin targets were prepared with 90 wt./wt.% distilled water mixed with the MNPs and 10 wt./wt.% gelatin. Furthermore, we produced 0% oil-gelatin and 40% oil-gelatin targets corresponding to the procedure presented by Lazebnik et al. [32] without the addition of formaldehyde. We analyzed three test glasses filled with distilled water as reference. The targets were positioned close to the magnetic pole by using a fixation, as shown in Figure 2b.

In the first step, we investigated the influence of the magnetic field intensity in the range of 0⋯140 kA/m on the MNP response for six targets (one target with distilled water, MNPs diluted in distilled water, MNPs embedded in agar, gelatin, and two types of oil-gelatin).

In the second step, we evaluated the differences caused by the surrounding medium of the MNPs. Therefore, we analyzed the MNP response at a magnetic field intensity of 80 kA/m. We prepared 18 targets and measured each of them 11 times resulting in a total number of 198 measurements.

### 2.4. MNP Differential Microwave Imaging Setup

**Figure 3:**
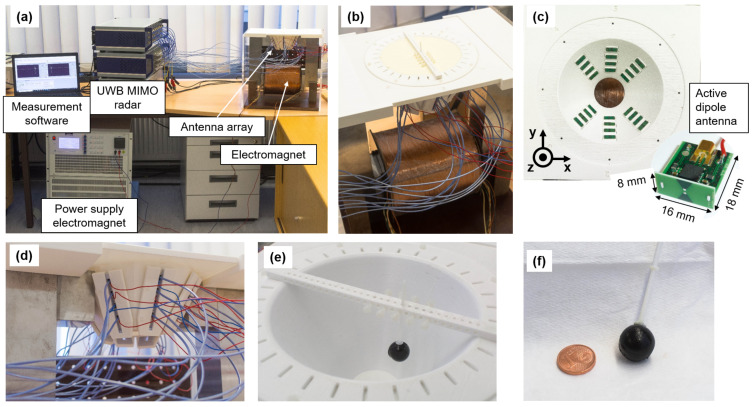
UWB microwave imaging setup via modulated MNPs: (**a**) Measurement setup. (**b**) Examination mold filled with the tissue-mimicking material (cream) and the target inside. (**c**) Top view of the antenna array showing the arrangement of the small active dipole antennas. (**d**) Antenna array in the air gap with the antenna attachment. (**e**) Examination mold with the tumor-mimicking target, which is positioned by means of a holder. (**f**) Target with a volume of 2 mL and an MNP concentration of 25 mg/mL.

The current imaging system was a further development of the system presented in our previous work [12,33]. Figure 3 shows the corresponding imaging setup. The electromagnet had an air gap of approximately 14 cm and was operated with a voltage source (EAC-S 3000, ET System electronic GmbH, Altlußheim, Germany). The antenna array with the active dipole antennas (see Figure 3c,d) and the examination mold (see Figure 3e) were placed inside the air gap. Additionally, we placed a silicone layer mixed with carbon between the antennas and the examination mold to ensure a better impedance matching. The antennas were connected to an UWB multiple-in and multiple-out (MIMO) radar system via nonmagnetic high-frequency cables. We used 8 transmitting and 16 receiving antennas, which resulted in a total number of 128 channels. The target was placed in the mold with the help of a holder, as shown in Figure 3e. The 2 mL tumor-mimicking target consisted of 10% gelatin mixed with distilled water and an MNP concentration of 25 mg/mL, as illustrated in Figure 3f. The surrounding medium was cream with a fat content of 32%, which represents healthy tissue (see Figure 3b). The dielectric properties of the cream in the frequency range between 1 GHz and 5 GHz can be found in the literature [34].

### 2.5. Signal Processing and Clutter Removal

**Figure 4:**
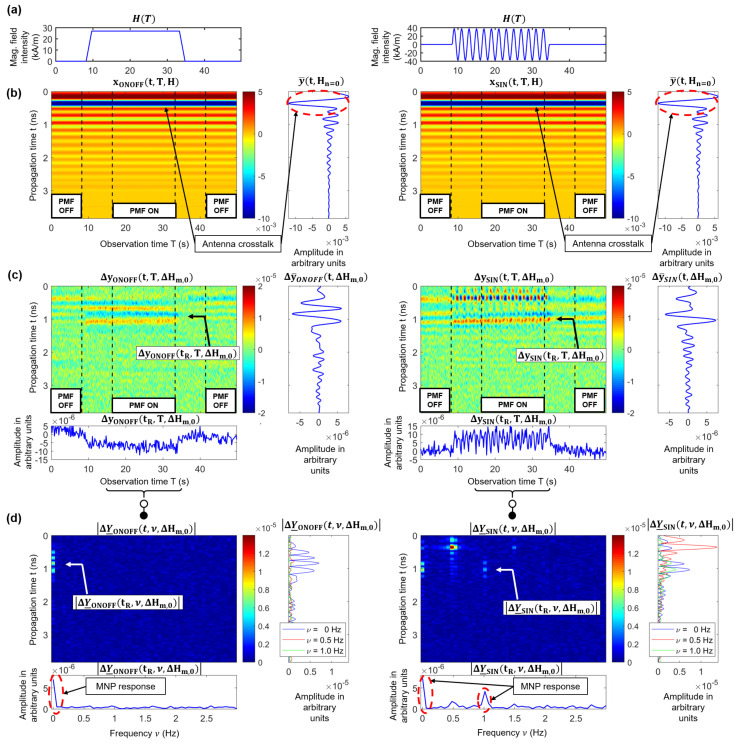
Exemplary signal processing and clutter removal for the ON/OFF modulation (left column) and the SIN modulation (right column): (**a**) Magnetic field intensity depending on the measurement time at the position of the MNPs. (**b**) Raw radargram $y_{MOD}\left(t,T,H\right)$ and the estimated clutter signal $\bar{y}\left(t,H_{n=0}\right)$ averaged over the time period without the presence of the external PMF. (**c**) Clutter removed radargram $\Delta y_{MOD}\left(t,T,\Delta H_{m,0}\right)$ with the mean MNP response $\Delta {\bar{y}}_{MOD}\left(t,\Delta H_{m,0}\right)$ averaged over the time period with the presence of the external PMF. (**d**) Single-sided magnitude spectrum $|\Delta {\underline{Y}}_{MOD}\left(t,\nu ,\Delta H_{m,0}\right)|$. The index $t_{R}$ represents the propagation time corresponding to the expected MNP response.

The detectability of MNPs is based on the change of the relative permeability of the MNPs by applying an external PMF, as described in Section 2.1. Figure 4 illustrates an exemplarily signal-processing procedure for an ON/OFF modulation, as well as for a sinusoidal modulation (SIN) of the PMF in order to extract the MNP response. The following equations are valid for both PMF modulation approaches where MOD indicates the type of modulation.

Figure 4a illustrates the magnetic field intensity H(T) at the position of the MNPs in the air gap depending on the observation time *T*. Between the time interval of two states (ON and OFF), the magnetic field was switched on or off via a ramp function to preserve the electromagnet. In addition, this time period was used to ensure that the magnetic field settled. Figure 4b shows the measured raw radargram $y_{MOD}\left(t,T,H\right)$, whereby each column corresponds to one measured IRF. In the first step, we estimate the clutter (e.g., antenna crosstalk), which superimposes the desired MNP response. It is calculated by averaging the IRFs over the time period without the presence of a PMF ($H_{n}=0$ kA/m) corresponding to:

(21) $$\bar{y}\left(t,H_{n}\right)=\frac{1}{T_{OFF}}\int_{T_{OFF}}y_{MOD}\left(t,T,H\right)\;dT,\;\;\;H_{n}=0\;\mathrm{kA}/m$$

where *t* is the propagation time, *T* the observation time (measurement time), and $T_{OFF}$ the interval in which the electromagnet is switched off (see Figure 4b). In the second step, the clutter removed radargram $\Delta y_{MOD}\left(t,T,\Delta H_{m,0}\right)$ (see Figure 4c) is computed by subtracting the mean IRF $\bar{y}\left(t,H_{n=0}\right)$ column by column from the raw radargram:

(22) $$\Delta y_{MOD}\left(t,T,\Delta H_{m,n}\right)=y_{MOD}\left(t,T,H_{m}\right)-\bar{y}\left(t,H_{n=0}\right)$$

and the mean MNP differential signal is computed by:

(23) $$\Delta {\bar{y}}_{MOD}\left(t,\Delta H_{m,0}\right)=\frac{1}{T_{ON}}\int_{T_{ON}}\Delta y_{MOD}\left(t,T,\Delta H_{m,0}\right)\;dT.$$

Due to the fact that the modulation stimulus of the PMF is known, the MNP response can be extracted in the frequency domain corresponding to the Fourier transform in observation time:

(24) $$\Delta {\underline{Y}}_{MOD}\left(t,\nu ,\Delta H_{m,0}\right)=\frac{1}{T_{ON}}\int_{T_{ON}}\Delta y_{MOD}\left(t,T,\Delta H_{m,0}\right)\cdot e^{-j2\pi \nu T}\;dT.$$

In the case of the ON/OFF modulation, the MNP response occurs at the DC component (ν=0 Hz), as illustrated in the left plot of Figure 4d. The MNP response of the periodic modulation ($\nu_{SIN}=0.5$ Hz) occurs at ν=0 Hz, and the second harmonic ν=1 Hz, as shown in the right plot of Figure 4d. The signal components at the first and third harmonics (0.5 Hz and 1.5 Hz) are related to the disturbing interactions of the PMF with the MWI system. It should be noticed that the MNP response shows a periodic shape corresponding to the absolute value of the SIN modulation, as depicted in the right plot ($\Delta y_{SIN}\left(t_{R},T,\Delta H_{m,0}\right)$) of Figure 4c. Due to the Fourier transform, the MNP response caused by the magnetic susceptibility modulation occurs at the even modulation harmonics of the PMF. This allows a separation between the MNP response and the slight disturbing influence exerted by the PMF at the odd harmonics, as described by Bucci et al. [35].

### 2.6. MNP Imaging

**Figure 5:**
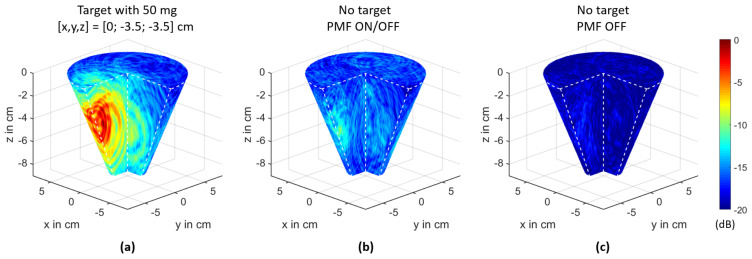
The 3D UWB images based on differential measurements with $\Delta y\left(t\right)=\Delta {\bar{y}}_{ONOFF}\left(t,\Delta H_{m,0}\right)$ (see Figure 4c) as the DAS input signal: (**a**) $I_{dB}\left(r_{0}\right)$ for the scenario with a 2 mL target with 50mg MNP at the position [x,y,z]=[0;−3;−3.5] cm. (**b**) $I_{dB,PMF}\left(r_{0}\right)$ for the scenario without a target and with the presence of a PMF. (**c**) $I_{dB,REF}\left(r_{0}\right)$ for the scenario without a target and no presence of a PMF. The images are normalized to the maximum intensity of Subplot (**a**).

The image processing is based on the delay-and-sum (DAS) beamforming algorithm. This qualitative imaging technique is appropriate for UWB imaging and is computed in the time domain, as described in the literature (e.g., [36]). The principle is based on the coherent summation of the backscattered radar signals after clutter removal. The volume of the MUT is divided into a grid of voxels. In order to obtain the intensity value *I* of the voxel located at position $r_{0}$, the signal components received from all antennas are superimposed with the propagation times according to the related transmitting antenna–voxel-receiving antenna distance. The absolute value of the clutter removed differential signals is used to compute the 3D UWB images according to:

(25) $$I\left(r_{0}\right)=\sum_{ch=1}^{N_{ch}}\left|\Delta y_{ch}\left(\tau_{ch}\left(r_{0}\right),\Delta H_{m,n}\right)\right|$$

where $N_{ch}$ is the number of channels, $r_{0}$ represents the coordinates of the focal point, and $\tau_{ch}\left(r_{0}\right)$ is the time delay from the transmitting antenna to the focal point and back to the receiving antenna of channel ch. The clutter removed differential signal of channel ch is represented by $\Delta y_{ch}$. We used 48 channels for image processing. The other ones were not included due to the fact that channel configurations with a wide angle between the transmitting and receiving antenna (e.g., transmission channels) do not improve the imaging quality, as investigated by Helbig et al. [37]. Figure 5 shows exemplarily the imaging results in decibels (dB) corresponding to:

(26) $$I_{dB}\left(r_{0}\right)=10\cdot \log_{10}\left(I^{2}\left(r_{0}\right)\right)$$

for the ON/OFF scenarios with a 2 mL target including 50 mg of MNPs (a) and without a target (b), as well as an image without a target and no presence of an external PMF (c). The scenario without a target and with the presence of a PMF shows differences compared to the image without a target and no presence of a PMF. These spurious effects are caused by the PMF and are considered in the following sections.

In order to evaluate the imaging results, we introduce a signal-to-clutter ratio:

(27) $$S/C=10\cdot log_{10}\left(\frac{\frac{1}{V_{target}}\int_{V_{target}}I^{2}\left(r_{0}\right)\;dV}{\frac{1}{V_{breast}}\int_{V_{breast}}I^{2}\left(r_{0}\right)\;dV}\right)$$

where the numerator is the mean intensity of the target volume and the denominator is the mean intensity of the breast volume without the target region.

### 2.7. Magnetic Field Analysis

The measurement setup presented in Section 2.4 shows an electromagnet with an air gap of 14 cm. In order to analyze the inhomogeneity of the magnetic field within the air gap, we measured the magnetic field intensity with a Gaussmeter (BGM 201, Dr. Brockhaus Messtechnik GmbH & Co. KG, Lüdenscheid, Germany). Due to the fact that an inhomogeneous magnetic field influences the MNP imaging results depending on the target position, it is essential to measure the magnetic field distribution within the air gap. These results can be used to correct the magnetic-field-dependent MNP response at different target positions. Therefore, we determined the magnetic field intensity $H(r_{0})$ for a total volume of [x×y×z]=[11×11×10] cm${}^{3}$ with a spatial resolution of 5 mm.

## 3. Results

### 3.1. Influence of Viscosity and Magnetic Field Intensity

**Figure 6:**
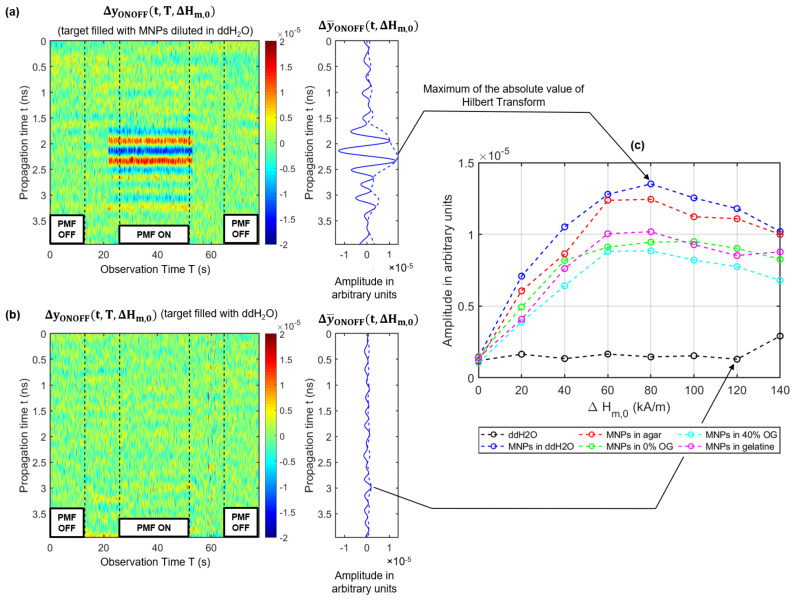
(**a**) Clutter removed radargram and the mean response for the target filled with MNPs diluted in distilled water. (**b**) Clutter removed radargram and the mean response for the reference target filled with distilled water. (**c**) Maximum of the envelope of the MNP responses as a function of the magnetic field intensity for different targets, whereby 0% OG and 40% OG indicate the mixture ratio of oil in gelatin corresponding to Lazebnik et al. [32]. The black curve corresponds to the reference measurement where the target is filled with distilled water without MNPs.

This section presents the results corresponding to the measurement setup described in Section 2.3. We investigated five targets of 15 mL in volume with MNPs embedded in different materials and one reference target. Figure 6 shows the radargrams after clutter elimination corresponding to Equation (22) for the target filled with MNPs diluted in distilled water (a) and for the reference target filled with distilled water (b). Figure 6c shows the maximum of the envelope of the MNP response for targets with different viscosities depending on the magnetic field intensity. The black curve indicates the results of the reference target filled with distilled water without MNPs and shows no dependency on the magnetic field intensity. The MNP responses of the MNP-loaded targets depend on the magnetic field intensity. The curves show a similar trend, where the MNP responses increase with increasing magnetic field intensity $H_{m}$ and reach their maximum in the range between 60 kA/m and 80 kA/m. The MNP responses decrease slightly for magnetic field intensities $H_{m}$ higher than 80 kA/m. Furthermore, the amplitudes of the curves vary depending on the surrounding medium in which the MNPs were embedded.

**Figure 7:**
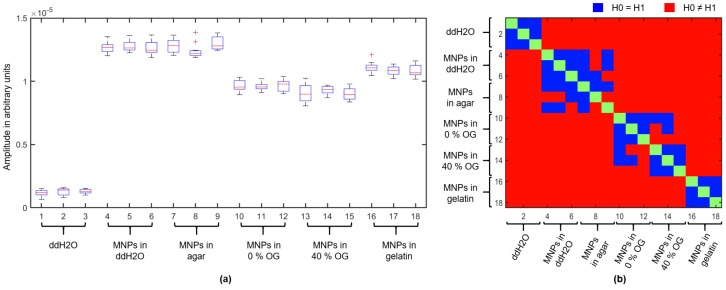
Results of the MNP response depending on the embedded medium, where 0% OG and 40% OG indicate the mixture ratio of oil in gelatin corresponding to Lazebnik et al. [32]. (**a**) Boxplots for each target showing the median (central mark of each box) and ±2.7 standard deviations (whiskers). (**b**) Results of the two-sample Kolmogorov–Smirnov test.

In order to examine these differences, we analyzed the MNP responses in a second experiment, where we measured the MNP responses eleven times for eighteen targets at a magnetic field intensity of $H_{m}=80$ kA/m. Figure 7a summarizes the maximum amplitudes of the envelope of the MNP responses where each boxplot represents the results for one target. The amplitudes are in accordance with the results shown in Figure 6c at $\Delta H_{m,0}=80$ kA/m and confirm a difference of the MNP response depending on the viscosity and surrounding medium, respectively. Furthermore, we compared the amplitude distribution of each target with all other targets by means of the two-sample Kolmogorov-Smirnov test [38]. This test examines whether the two datasets of the different targets have the same distribution. Figure 7b shows the results, whereby the test confirms the null hypothesis H0=H1 (both datasets are from the same continuous distribution) at the 5% significance level and otherwise rejects the null hypothesis H0≠H1. The test shows a significant difference between the reference measurement (pure distilled water) and all other targets with MNPs, which means that the MNPs can be detected in all mixtures. Furthermore, it can be distinguished significantly between three “test groups” of targets. Group 1 consists of the test glasses with MNPs diluted in distilled water and embedded in agar; Group 2 includes the test glasses filled with the MNPs embedded in oil-gelatin according to Lazebnik et al. [32]; Group 3 represents the targets with MNPs embedded in 10% gelatin. However, the results show no significant differences between the magnetite nanoparticles diluted in distilled water and embedded in agar. Furthermore, we can not distinguish unambiguously between MNPs embedded in 0% or 40% oil-gelatin.

### 3.2. MNP Imaging

**Figure 8:**
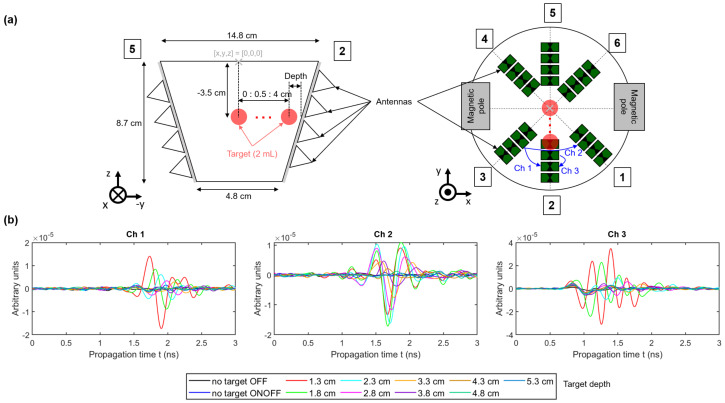
(**a**) Schematic of the measurement setup and the different target positions. (**b**) Differential IRFs for different channels with the depth-dependent MNP response. The depth is related to the distance between the target and the wall of the examination mold.

This section investigates the different aspects of MNP imaging corresponding to the setup presented in Section 2.4. First, we analyzed the influence of the target’s position on the MNP response. Figure 8a shows the schematic of the measurement setup with the corresponding target positions. The target had a volume of 2 mL consisting of 10% gelatin mixed with distilled water and 50 mg MNPs. We measured the MNP responses at nine positions along the y-axis in steps of 5 mm, as illustrated in Figure 8a. Furthermore, three channels with the MNP responses $\Delta {\bar{y}}_{ONOFF}\left(t,\Delta H_{m,0}\right)$ for different target depths are shown in Figure 8b. The responses decrease with rising depth for Channel 1 and Channel 3, whereby the latter one has the highest amplitude due to the short distance from the transmitting and receiving antenna to the target. However, Channel 2 has the highest amplitude for a target depth of 1.8 cm, which can be explained by the angle-dependent radiation pattern of the dipole antennas, as investigated by Helbig et al. [39].

**Figure 9:**
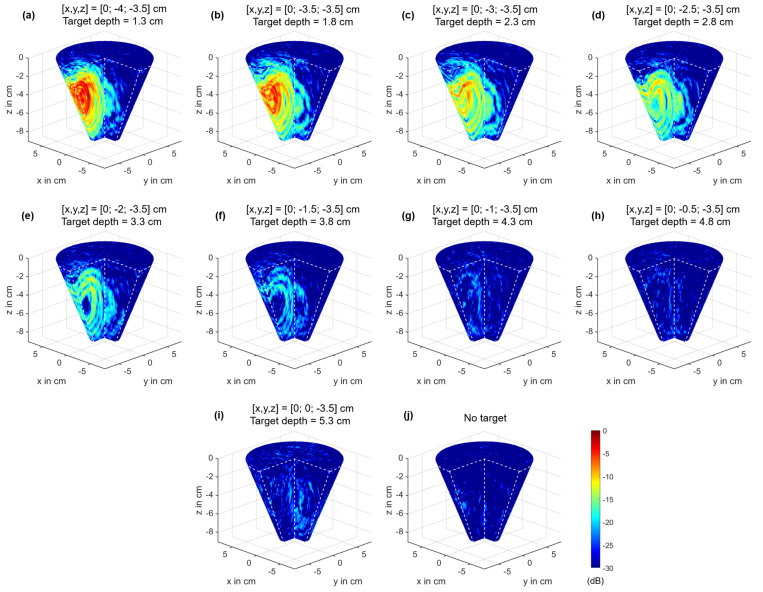
Imaging results of the 2 mL target with an MNP concentration of 25 mg/mL for different target positions. The images are normalized to the maximum intensity of Subplot (**a**).

Figure 9 shows the imaging results of the 2 mL target for the different target positions. The spurious effects, which occur during a measurement with the presence of a PMF, as illustrated in Figure 5, are eliminated by an image subtraction corresponding to:

(28) $$I_{CF_{PMF}}\left(r_{0}\right)=I_{MNP}\left(r_{0}\right)-I_{PMF}\left(r_{0}\right)$$

where $I_{MNP}\left(r_{0}\right)$ is the image with MNPs and $I_{PMF}\left(r_{0}\right)$ is the reference measurement without MNPs. Both images are computed by Equation (25) with the DAS input signal $\Delta y\left(t\right)=\Delta {\bar{y}}_{ONOFF}\left(t,\Delta H_{m,0}\right)$. Finally, the images $I_{CF_{PMF}}\left(r_{0}\right)$ presented in Figure 9 are calculated according to Equation (26).

**Figure 10:**
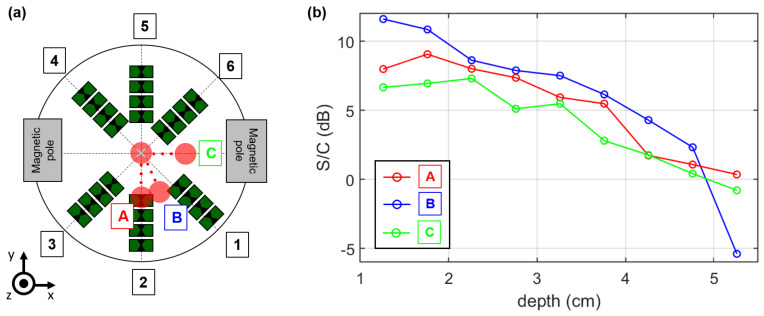
(**a**) Schematic of the antenna array and the target positions. (**b**) Corresponding signal-to-clutter ratio (S/C) depending on the target depth for the three different axes A, B, and C.

We repeated the depth-dependent measurements for two additional axes, as illustrated by the schematic in Figure 10a. Furthermore, we computed the S/C ratio by Equation (27) depending on the target depth in order to analyze the detectability of the MNPs (see Figure 10b). The S/C ratio decreases with increasing depth and shows that it is possible to detect the MNPs until a depth of approximately 4.3 cm. At greater distances, the S/C ratio is close to zero or less, which indicates that the tumor do not raise above the clutter.

### 3.3. Magnetic Field Influence

**Figure 11:**
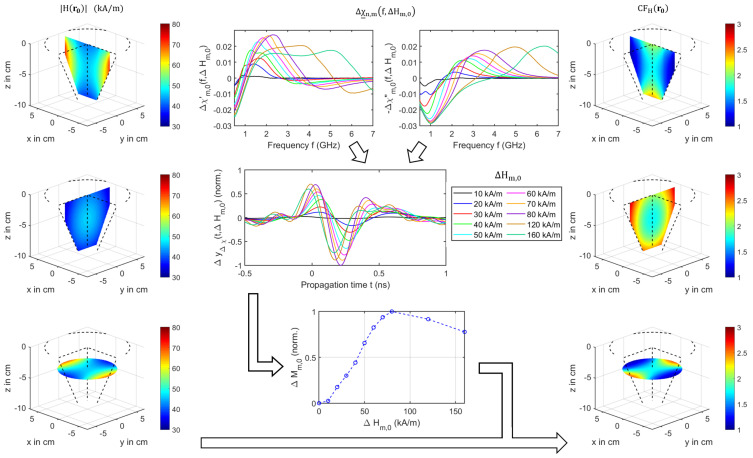
Compensation of the magnetic field inhomogeneity. The absolute value of the measured magnetic field intensity $\left|H(r_{0})\right|$ (left column). Differential magnetic susceptibility $\Delta {\underline{\chi }}_{m,0}\left(f,H_{m,0}\right)$ depending on the frequency for different magnetic field intensities (according to [40]), the estimated differential signal $\Delta y_{\Delta \chi }\left(t,\Delta H_{m,0}\right)$, and the derived normalization curve as a function of $\Delta H_{m,0}$ with $B_{f}=$1–4 GHz scaled to its maximum at $H_{m,0}=80$ kA/m (middle column). The 3D correction matrix $CF_{H}\left(r_{0}\right)$ computed by Equation (31) (right column).

The results presented in Section 3.1, as well as given by the literature [31,40] demonstrate that an inhomogeneous magnetic field influences the imaging results of the MNPs. The curves show a nonlinear behavior between the magnetic field intensity and the MNP response with respect to the working frequency range of the MWI system. In order to compensate the nonlinearity, we derive a correction factor from Bellizzi et al. [40]. Figure 11 illustrates the compensation approach, where the results of our magnetic field intensity measurements $\left|H(r_{0})\right|$ (see Section 2.7) are shown exemplarily for one x-, y-, and z-plane on the left-hand side. Furthermore, the differential magnetic susceptibility $\Delta {\underline{\chi }}_{m,n}\left(f,\Delta H_{m,n}\right)$ as given by Bellizzi et al. [40] is depicted. The knowledge of both parameters can be exploited to estimate a magnetic-field-intensity-dependent normalization factor corresponding to:

(29) $$\Delta M_{m,n}=\max\left|\Delta y_{\Delta \chi }\left(t,\Delta H_{m,n}\right)\right|=\max\left|\int_{B_{f}}\Delta {\underline{\chi }}_{m,n}\left(f,\Delta H_{m,n}\right)e^{j2\pi ft}\;df\right|$$

whereby $B_{f}$ determines the working frequency range (1–4 GHz) and $H_{n}=0$ kA/m due to the ON/OFF measurement scenario. The differential magnetic susceptibility is defined by:

(30) $$\Delta {\underline{\chi }}_{m,n}\left(f,\Delta H_{m,n}\right)=\underline{\chi }\left(f,H_{m}\right)-\underline{\chi }\left(f,H_{n}\right).$$

The data of $\underline{\chi }\left(f,H\right)$ for the MNPs under consideration can be found in Bucci et al. [28]. Figure 11 (middle) shows the results of the estimated differential signal $\Delta y_{\Delta \chi }\left(t,\Delta H_{m,0}\right)$ corresponding to Equation (29). The curves are normalized to the maximum value at $\Delta H_{m,0}=80$ kA/m. Please note that we focus on the change of the magnetic properties, so in contrast to Bellizzi, the normalization factor is independent of the channel-dependent path. Therefore, the transfer function of the MUT with respect to the dielectric properties over the working frequency range is constant. In addition, Figure 11 (middle) depicts the nonlinear behavior between the absolute maximum of the estimated differential signal $\Delta M_{m,n}$ and the magnetic field intensity. Finally, the 3D correction matrix is computed by:

(31) $$CF_{H}\left(r_{0}\right)=\frac{1}{\Delta M_{m,n}\left(r_{0}\right)},\;\;\;\;\;\Delta M_{m,n}\neq 0$$

whereby $CF_{H}\left(r_{0}\right)$ is the magnetic-field-dependent correction factor, which is illustrated in the right column in Figure 11 for three different planes.

**Figure 12:**
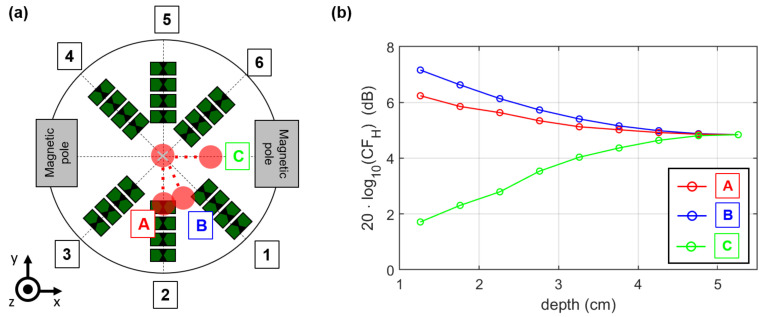
(**a**) Schematic of the antenna array and the target positions. (**b**) Correction factor $20\cdot \log_{10}\left(CF_{H}\right)$ in decibels (dB) depending on the target position.

In order to analyze the effect of the correction step, we considered the correction factor at different target positions, as shown in Figure 12. The correction of the magnetic field influence on the MNP response indicates the highest values along the A- and B-axes, where the distance between the tumor and the wall is low. This is in accordance with the magnetic field measurements, because the magnetic field intensities are low along these axes. The correction amplitude decreases with increasing depth towards the center of the examination mold. In contrast, the correction of the target along the C-axis showes the lowest values due to the high magnetic field intensity close to the magnetic pole. Here, the correction amplitude increases with growing distance between the target and the magnetic pole. In terms of MNP imaging, Equation (25) can be extended by the correction factor:

(32) $$I_{CF_{H}}\left(r_{0}\right)=CF_{H}\left(r_{0}\right)\cdot \sum_{ch=1}^{N_{ch}}\left|\Delta y_{ch}\left(\tau_{ch}\left(r_{0}\right),\Delta H_{m,n}\right)\right|$$

to compensate the inhomogeneity of the magnetic field distribution. Without taking the other influencing factors (e.g., path-dependent attenuation) into account, such a correction leads to the same intensity of an MNP-loaded target in the UWB image independent of its position and the magnetic field intensity distribution, respectively.

### 3.4. PMF Modulation

**Figure 13:**
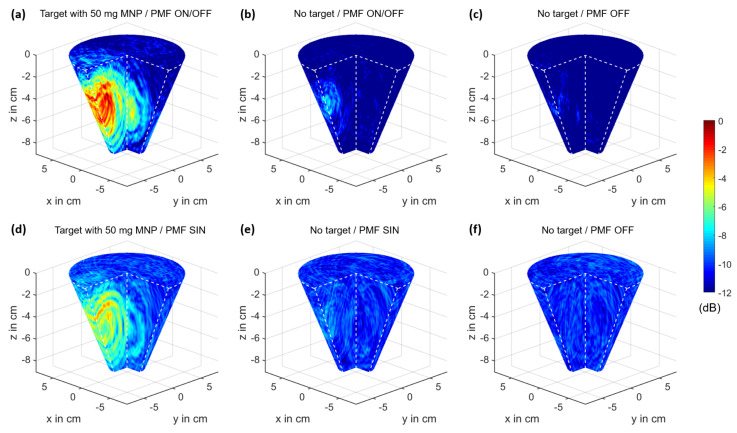
The 3D UWB images based on ON/OFF and SIN modulation of the PMF, as well as no presence of a PMF corresponding to Equations (25) and (26). DAS beamforming with the DC component $|\Delta {\underline{Y}}_{ONOFF}(t,\nu =0$ Hz, $\Delta H_{m,0})|$ (**a**,**b**) and $|\Delta {\underline{Y}}_{OFF}(t,\nu =0$ Hz, $\Delta H_{0,0})|$ (**c**) as the input signal, as well as the second harmonic $|\Delta {\underline{Y}}_{SIN}(t,\nu =1$ Hz, $\Delta H_{m,0})|$ (**d**,**e**) and $|\Delta {\underline{Y}}_{OFF}(t,\nu =1$ Hz, $\Delta H_{0,0})|$ (**f**) as input signal. (**a**,**d**) The 2 mL target with a concentration of 25 mg/mL at position [x,y,z]=[0;−3;−3.5] cm. (**b**,**e**) ON/OFF and SIN modulation, respectively, without a target. (**c**,**f**) No presence of a PMF and without a target. The images are normalized to the maximum intensity of Subplot (**a**).

In comparison to the ON/OFF modulation, a periodic PMF modulation is a promising approach to separate system drift effects (caused, e.g., by weak temperature variations) and disturbing influences of the PMF on the setup from the desired MNP response. Therefore, we analyzed the MNP response for both modulation types. The 2 mL target with an MNP concentration of 25 mg/mL was placed at the position [x,y,z]=[0;−3;−3.5] cm. We adjusted the magnetic field intensity at the target position to the same RMS value ($H_{RMS}=27$ kA/m) for both modulations to ensure the comparability. The sampling rate is approximately six IRFs per second, and the 3D UWB images are computed in decibels (dB) corresponding to Equations (25) and (26) with $\Delta y\left(t\right)=|\Delta {\underline{Y}}_{MOD}\left(t,\nu ,\Delta H_{m,0}\right)|$ for ν=0 Hz or ν=1 Hz as the DAS input signal. Figure 13a,d shows the imaging results for the two-state modulation and the sinusoidal modulation of the PMF with an MNP-loaded target. In addition, we investigated the disruptive influence of the PMF on the imaging results for both types of modulation. Therefore, we computed reference images without a target, but with the presence of the external PMF (see Figure 13b,e) and without the presence of an external PMF (see Figure 13c,f). The images with the MNP-loaded target show higher intensities compared to the images without a target, whereby the ON/OFF modulation has the highest intensity. However, the image of the ON/OFF modulation without a target shows different intensities compared to the corresponding image without the presence of a PMF, which indicates a slight influence of the PMF on the measurements. In contrast, the image with the SIN modulation without a target shows the same intensities compared to the corresponding image without the external PMF.

Based on the images shown in Figure 13, we computed the S/C ratio as introduced in Section 2.6 to compare both approaches. The results are summarized in Table 1. Considering the ON/OFF modulation, it is obvious that the S/C ratio shows the highest value for the scenario with MNPs at the DC component (ν=0 Hz). However, this also indicates a slight influence of the PMF on the measurements, because the S/C ratios differ between the scenarios (no target) with and without the presence of the external PMF. For the sake of completeness, we also calculated the S/C ratios at the second harmonic. As expected, the ratios show low values, indicating no MNP response, as well as no disturbing influences.

Concerning the SIN modulation, the S/C ratios also show the highest value for the scenario with MNPs at the DC component, but a slight spurious influence is also present, as shown by the difference in the S/C ratio between the SIN modulation without a target and the OFF measurement without a target. In addition, the SIN modulation shows an MNP response at the second harmonic (ν=1 Hz). In contrast to the DC component analysis, the S/C ratios at this harmonic do not differ between the SIN modulation without a target and the OFF measurement without target, providing an efficient separation between the clutter components and the desired MNP response.

## 4. Discussion

In the first experiment, we analyzed the influence of the magnetic field intensity and the viscosity of different targets on the MNP response. Bellizzi et al. [40] figured out that there is a nonlinear dependency between the magnetic field intensity of the PMF and the MNP response with respect to the bandwidth of our UWB radar system. Furthermore, they determined the optimal magnetic field intensities of $H_{m}=80$ kA/m and $H_{n}=0$ kA/m in terms of the ON/OFF modulation based on the complex magnetic susceptibility of the MNPs dissolved in phosphate buffer saline [28,40]. These findings are in accordance with our results for the MNPs diluted in distilled water, as shown in Figure 6. Furthermore, the nonlinear dependency of the MNP response on the magnetic field intensity is also valid when the MNPs are embedded in a solid medium. All targets with MNPs show a similar curve shape with the maximum amplitude of the MNP response at approximately $\Delta H_{m,0}=80$ kA/m, since the difference in the magnetic susceptibility of the MNPs for $\Delta H_{m,n}$ with $H_{m}=80$ kA/m and $H_{n}=0$ kA/m related to the working frequency range of our radar system (≈1–4 GHz) becomes a maximum. The corresponding measurements of the magnetic field dependent magnetic susceptibility of the MNPs as a function of frequency are given by Bucci et al. [28] and Bellizzi et al. [40]. However, the plots show differences in their amplitudes, which is confirmed by the results of a further experiment, as depicted in Figure 7. While the targets with MNPs diluted in distilled water and embedded in agar show no significant differences, which is consistent with our preliminary measurements [31], the targets with MNPs embedded in gelatin and oil–gelatin show a lower response. Embedding the MNPs in a solid surrounding material leads to the immobilization of the particles, which means that Brownian relaxation is eliminated. Since the Brownian relaxation influences the susceptibility in the lower frequency range, as described in Section 2.1, this cannot be the reason for the different amplitudes of the MNP responses. Rather, the dielectric properties of the targets influence the results, because the different complex permittivities of distilled water, agar, gelatin, and oil–gelatin result in different reflection coefficients at the boundary between the target and the surrounding medium, as described in Section 2.1 and indicated by Equations (15)–(20). A further aspect could be the low amount of n-propanol in the oil-gelatin targets corresponding to the mixtures presented by Lazebnik et al. [32]. Mixing the MNPs with pure n-propanol results in a clumping of the particles. It might be possible that the low amount of n-propanol of the 0% and 40% oil-gelatin materials leads to small MNP clusters, which influence their susceptibility and lower the MNP response.

The second part of the paper dealt with our MNP imaging system. We analyzed the MNP response depending on the target position. Therefore, we used a tumor substitute consisting of 10% gelatin due to the results of Section 3.1. Gelatin is a more suitable substitute compared to distilled water due to the lower permittivity and due to the binding of the MNPs to gelatin in terms of their immobilization, as shown by Dutz et al. [41]. Section 3.2 presents the results of the imaging procedure for a 2 mL target with an MNP concentration of 25 mg/mL. Figure 9 shows that the intensity of the tumor decreases with increasing target depth, whereby it is still detectable at a depth of 4.3 cm, as indicated by the S/C ratio. For greater depths, the ratio becomes close to or less than zero, which indicates that the MNP response do not stand out from the clutter, as depicted in Figure 10. The detectable depth is valid for the presented measurement scenario. Due to the fact that we used phantom materials with respect to the dielectric properties of biological tissue, we conclude that such amounts of MNPs can be also detected in in vivo measurements up to a depth of about 4 cm.

The results offer some challenges related to MNP imaging. We investigated the imaging results for a tumor imitate with a constant volume and magnetite concentration at different positions, and we see variations of the S/C ratio depending on both the target depth and the investigated axes (A,B,C). These differences make it difficult to extract more information from the UWB images (e.g., amount of MNPs or the target size). The main reason that the MNP response decreases with increasing target depth is the path-dependent attenuation of the electromagnetic wave, which is caused by the surrounding medium (cream). Furthermore, the antennas cannot be arranged uniformly due to the magnetic poles. This results in an inhomogeneous illumination of the MUT by the antennas, which lowers the S/C ratio if the target is located close to the wall of the examination mold and between two antenna slots (e.g., at the B- and C-axes). This becomes evident if we consider the S/C ratio along the B- and C-axes, where the maximum is reached at a depth of 1.8 cm and 2.3 cm, respectively (see Figure 10b) due to the angle-dependent radiation pattern of the antennas as described in Section 3.2 and exemplarily shown for Channel 2 in Figure 8b.

The MNP responses and the imaging results are also influenced by the inhomogeneity of the magnetic field. In order to compensate this influence, we presented a correction approach based on the measured magnetic field intensity in the air gap of the electromagnet and the magnetic susceptibility of the MNPs in order to image a target with the same intensity independent of the magnetic field distribution. Such a compensation only makes sense if an MNP response can be detected reliably. Considering a practical measurement scenario, the disruptive influence of the PMF on the MWI system has a negative effect on a robust MNP detection and imaging, respectively, as shown exemplarily in Figure 5. With regard to a reliable detection, it is necessary to suppress or to separate the disruptive influence. Otherwise, it becomes difficult to distinguish whether a change is caused by the spurious effects of the PMF or by the MNPs. Therefore, we estimated the disruptive influence in terms of the ON/OFF modulation by a differential imaging without MNPs, but with the presence of the PMF. In a practical measurement scenario, this means that we acquire a reference differential image before the MNPs are injected intravenously. Afterwards, the MNPs are injected, and a second measurement is performed after a sufficient amount of MNPs has bound to the tumor. Since there are two UWB differential images (with and without MNPs), the disturbing influence of the PMF should be comparable in both cases and can be reduced by subtracting both images according to Equation (28), even after the breast has been repositioned.

**Figure 14:**
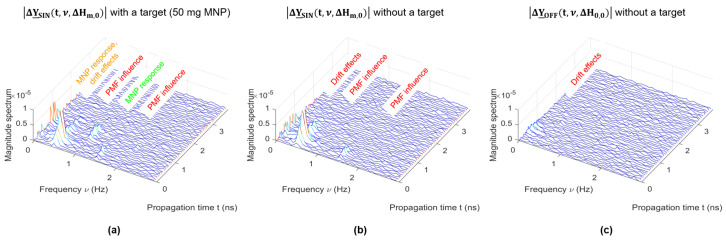
Fourier transform over observation time of the clutter removed radargrams according to Equation (24) of one channel for different scenarios. (**a**) SIN modulation with a 2 mL target with an MNP concentration of 25 mg/mL. (**b**) SIN modulation without a target. (**c**) Without a target and no presence of an external PMF.

Due to the fact that differential imaging including breast repositioning will be affected by repositioning errors, a further promising approach is the periodic modulation of the PMF, as described in Section 3.4. The sinusoidal modulation of the PMF enables a separation of the disturbing influence of the PMF from the MNP response. Figure 14a shows the Fourier transform of the clutter removed radargram corresponding to Equation (24) of one channel with an MNP-loaded target. The radargram has different signal components, whereby the MNP response corresponds to the second harmonic (ν=1 Hz) and the DC component (ν=0 Hz). However, the latter component is overlapped by a disturbing influence caused by drift effects. This is evident due to the results of Figure 14b,c, where we also see a low signal component at the DC value (ν=0 Hz) even though there is no target. Considering Figure 14a,b, the spurious effects occur at the modulation frequency of 0.5 Hz and the third harmonic in both scenarios, with and without a target. The reason for this influence is related to the interactions of the PMF on the MWI system, as described by Bucci et al. [17]. Furthermore, they figured out that spurious effects that occurred at the second harmonic are associated with hidden magnetic materials in the measurement setup [17,35]. Since we used nonmagnetic cables and connectors, such disturbing signal components were suppressed in our measurements, as illustrated by Figure 14b. In addition, Figure 14c shows the radargram without a target and no presence of a PMF where only minor drift effects occur in the low-frequency range. These effects are related to weak and slow temperature variations of the measurement hardware and can be separated from the MNP response with a sufficiently high modulation frequency of the PMF. If the MNP response at the second harmonic is used as the input signal for the imaging process, the difference between the reference measurement (no target and no PMF) and the PMF measurement (no target and SIN modulation) will be quiet low, as illustrated in Figure 13e,f, and confirmed by the S/C ratio, as shown in Table 1. In this case, the MNPs can be detected reliably. In comparison, the ON/OFF modulation shows higher intensities compared to the SIN modulation, as depicted in Figure 13a,d, and the PMF can be chosen with respect to the optimal magnetic field intensities of $H_{m}=80$ kA/m and $H_{n}=0$ kA/m. This means that lower amounts of MNPs can be detected with the ON/OFF modulation than with the SIN modulation. However, the two-state modulation exhibits a slight disruptive influence caused by the PMF, which can be also interpreted as the MNP response (see Figure 13b). Therefore, an additional measurement (before MNP injection) is necessary in order to estimate this influence, which is not the case in terms of the sinusoidal modulation technique due to the separation of the different signal components.

## 5. Conclusions

In this contribution, we investigated different aspects of MNP detection and imaging by means of UWB pseudo-noise sensing. First, we could demonstrate that the measured MNP response depends on the magnetic field intensity of the external PMF for MNPs diluted in distilled water, as well as embedded in a solid medium. The relationship between the MNP response and magnetic field intensity is nonlinear with respect to the working frequency range of the MWI system. Therefore, it is crucial to choose the optimal magnetic field intensities to increase the detectability of the MNPs. In conclusion, we can say that immobilized MNPs can be detected by means of UWB sensing, which is important due to the study of Dutz et al. [41], where the immobilization of the MNPs in the tumor tissue was demonstrated.

In the second part of this paper, we presented a realistic UWB measurement setup for contrast-enhanced breast cancer imaging. We could demonstrate that it is possible to image a 2 mL target with an MNP concentration of 25 mg/mL up to a depth of approximately 4.3 cm with this setup. In order to detect MNPs at a greater depth, the contrast can be enhanced by increasing the magnetic field intensity in the air gap up to the optimal magnetic field intensity of 80 kA/m. Furthermore, an increased measurement time results in a higher number of measured IRFs, which can be used to reduce the noise level due to the averaging procedure. Both methods (higher magnetic field intensity and longer measurement time) cause an increased heating of the electromagnet’s coil. For this reason, suitable measures must be implemented to counteract this heating process.

Furthermore, we compared the ON/OFF and sinusoidal PMF modulation with regard to the MNP detection and the spurious effects caused by the interactions of the PMF and the MWI system. Regarding the latter aspect, we presented an approach to lower the disturbing influence for the ON/OFF, as well as for the SIN modulation.

Finally, we can conclude that the MNPs can be successfully detected with both modulation types. However, we see that the MNP response depends on the target position. In this work, we presented an approach to compensate the magnetic-field-dependent influence on the MNP response with respect to the inhomogeneous magnetic field distribution. In future work, we plan to extend the method in terms of compensating the influence of dielectric properties (e.g., path-dependent attenuation) to obtain the MNP response independent of the target position and the surrounding tissue. Furthermore, it is necessary to detect even smaller amounts of MNPs and to apply the PMF modulation techniques in in vivo measurements.

## Figures and Tables

**Table 1:** S/C ratio for ON/OFF and SIN modulation, as well as for the scenario without the presence of an external PMF.

PMF	ON/OFF		SIN ($\nu_{\mathrm{SIN}}=0.5$ Hz)		OFF
Target	50 mg (2 mL)	No Target	50 mg (2 mL)	No Target	No Target
ν=0 Hz	5.7 dB	1.8 dB	4.9 dB	2.7 dB	0.9 dB
ν=1 Hz	−0.4 dB	−0.3 dB	3.4 dB	0.1 dB	0 dB

## Data Availability

Data sharing is not applicable.

## Abbreviations

The following abbreviations are used in this manuscript:

3D	Three-dimensional
DAS	Delay-and-sum
DC	Direct current
IRF	Impulse response function
MIMO	Multiple-in and multiple-out
MNPs	Magnetic nanoparticles
MPI	Magnetic particle imaging
MRI	Magnetic resonance imaging
MRX	Magnetorelaxometry
MUT	Medium under test
MWI	Microwave imaging
OG	Oil–gelatin
PMF	Polarizing magnetic field
UWB	Ultra-wideband
